# Eslicarbazepine acetate is porphyrogenic and should be used with caution in patients with the acute hepatic porphyrias

**DOI:** 10.3389/fphar.2022.953961

**Published:** 2022-09-06

**Authors:** Christopher D. Ma, Herbert L. Bonkovsky

**Affiliations:** Department of Internal Medicine, Section on Gastroenterology and Hepatology, Wake Forest University School of Medicine, Winston-Salem, NC, United States

**Keywords:** porphyrogenicity, acute hepatic porphyria, porphyric attack, eslicarbazepine acetate, AEDs

## Abstract

Eslicarbazepine acetate, a third-generation antiepileptic drug (AED), has shown improved clinical response and safety in comparison to older generation AEDs for patients with partial-onset seizures. It is currently not known whether eslicarbazepine acetate is safe to use in patients with the acute hepatic porphyrias (AHPs) since a few first-generation AEDs, such as phenobarbital and carbamazepine, are known porphyrogenic agents. In this study, we used a recently published *in vitro* fluorescence-based screening assay to screen for porphyrogenicity in various agents. The assay confirmed that among the tested compounds used, allyl isopropyl acetamide, carbamazepine, eslicarbazepine acetate, and phenobarbital were porphyrogenic. Thus, eslicarbazepine acetate should be avoided if possible in patients with the AHPs, but if initiated, patients should be closely monitored and the drug should be discontinued if a porphyric exacerbation occurs.

## Introduction

Epilepsy is one of the most common neurological conditions in the world, and antiepileptic drugs (AEDs) have improved the quality of life of millions of epileptic patients (Hanaya and Arita, 2016). As for many drug classes, newer AEDs have been developed to improve clinical response and safety while decreasing adverse effects and drug-drug interactions. Eslicarbazepine acetate, a third-generation AED with a similar structure to the first-generation AED, carbamazepine, has shown promising results with a safer side-effect profile in treating partial-onset seizures (Galiana et al., 2017). Carbamazepine, for example, is a potent inducer of multiple cytochrome P450 (CYP) enzymes, such as CYP1A2, CYP2C9, CYP2C19, CYP3A4, and CYP3A5, which can subsequently increase the demand for heme biosynthesis (Lynch and Price, 2007; Correia et al., 2011). This can be dangerous for patients with the acute hepatic porphyrias (AHPs), which are diseases where one of the enzymes in the heme biosynthesis pathway is deficient, and in which increased heme demand in hepatocytes leads to upregulation of hepatic delta-aminolevulinic (ALA) synthase-1, the first and normally rate-controlling step of this pathway. This can lead to the biochemical hallmark and *sine qua non* of acute porphyric attacks, namely, markedly elevated levels of ALA and porphobilinogen (PBG). Eslicarbazepine acetate, although it belongs to the same dibenzazepine carboxamide family as carbamazepine, has been reported to be a weaker inducer of CYP3A4, to have a lesser inhibitory effect on CYP2C19, and overall, fewer enzymatic interactions than carbamazepine (Galiana et al., 2017). Thus, it is thought that eslicarbazepine acetate may prove safer to use in patients with the AHPs; (Herrera-Fortin et al., 2020) however, it is currently not known whether it is porphyrogenic or not. In this study, we assessed the porphyrogenicity of eslicarbazepine acetate and a few other drugs with a recently published *in vitro* fluorescence-based screening assay in Leghorn Male Hepatoma (LMH) cells, which is a hepatocellular carcinoma cell line derived from male leghorn chickens treated with diethylnitrosamine (Kawaguchi et al., 1987; Kolluri et al., 1999).

## Methods

**FIGURE 1:**
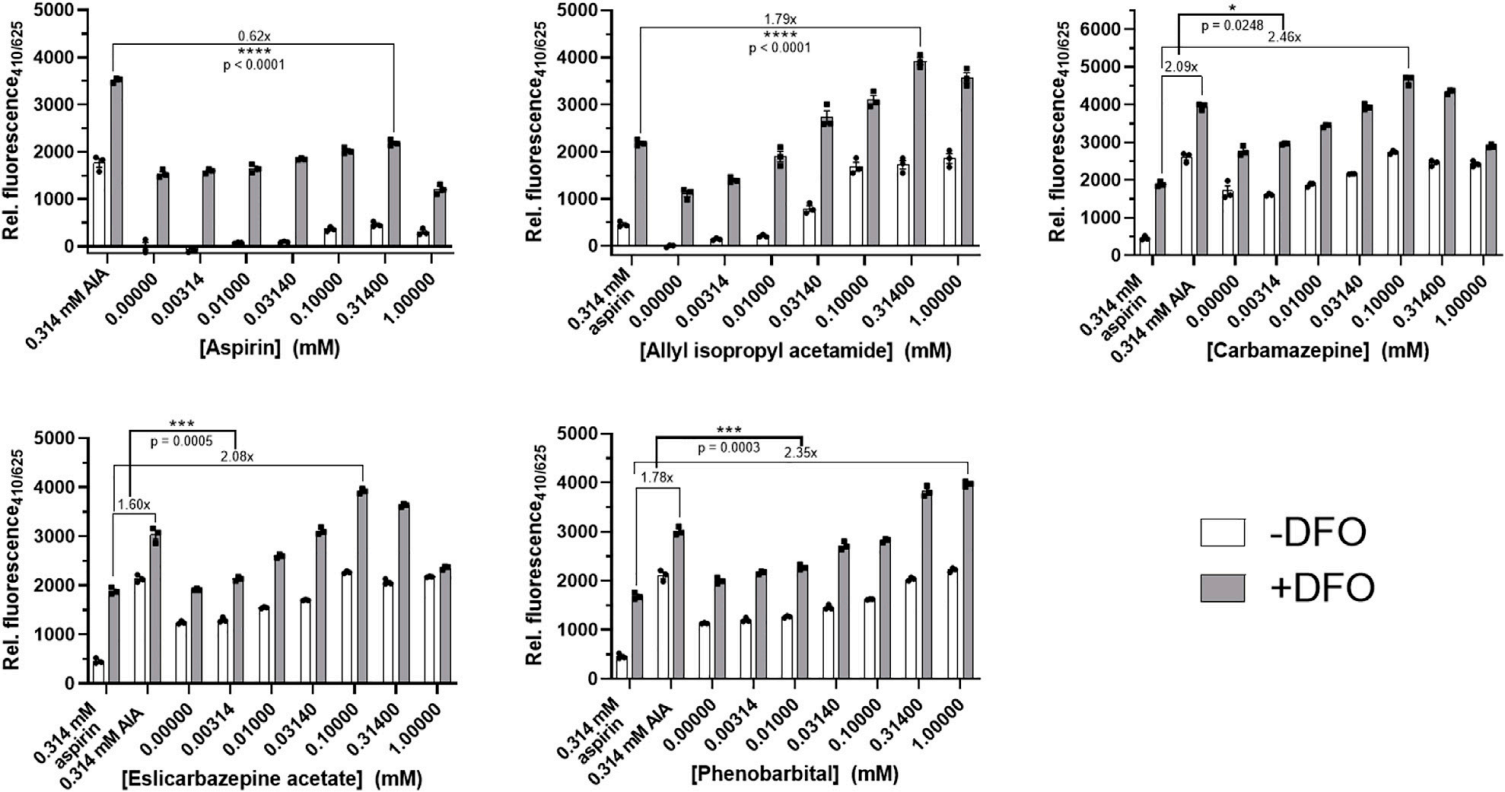
Porphyrogenicity of eslicarbazepine acetate and other compounds in LMH cells. Deferoxamine (DFO) is an iron chelator that prevents the conversion of the fluorescent intermediate, protoporphyrin, into heme. DFO is added in all experimental groups to mimic the conditions of the AHPs and to increase fluorescence measurements read by the fluorospectrometer. 0.314 mM aspirin and allyl isopropyl acetamide were negative and positive controls, respectively, and were added in all experiments to distinguish porphyrogenicity from non-porphyrogenicity in selected compounds. Higher concentrations of carbamazepine, eslicarbazepine acetate, and phenobarbital produced fluorescence readings greater than the readings produced by 0.314 mM AIA while all concentrations of aspirin produced readings below the readings produced by AIA. All data are presented as mean values ±SEM of three independent replicates. All results are representative of three independent experiments. A two-sided Student’s *t*-test was used to assess statistical significance.

**FIGURE 2:**
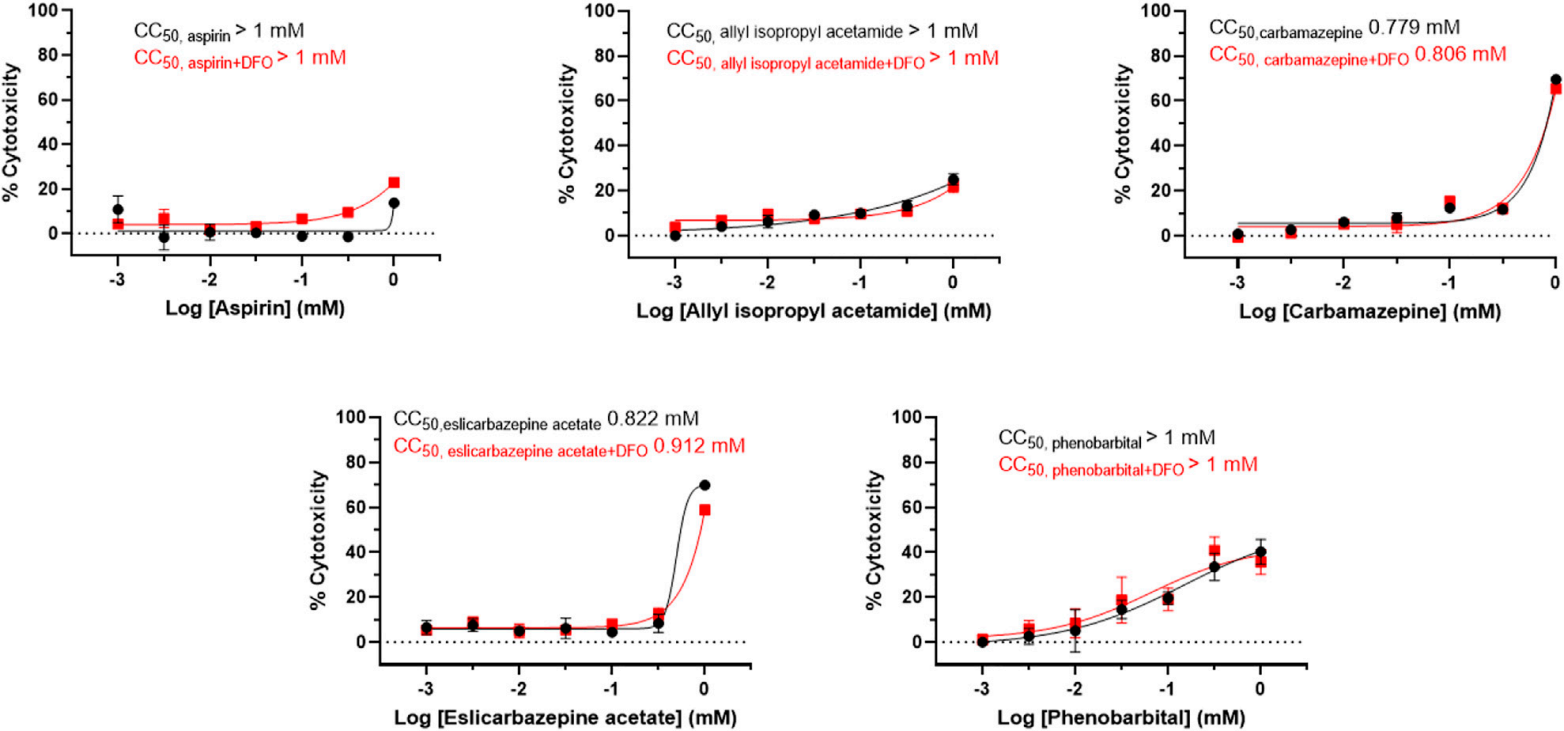
Cytotoxicity of eslicarbazepine acetate and other compounds in LMH cells. The ATPLite cytotoxicity assay was completed in parallel with the drug screening assays. CC_50_ values were calculated using the software, GraphPad Prism 8. All data are presented as mean values ± SEM of three independent replicates. All results are representative of three independent experiments.

The methods for the fluorescence-based screening assay and the cytotoxicity assay are described in Ma et al. (2022). The LMH cells were purchased from ATCC (Manassas, VA, United States) and maintained in Waymouth medium (Thermo Fisher Scientific, Waltham, MA, United States) supplemented with penicillin-streptomycin and fetal bovine serum. LMH cells were seeded (1 × 10^4^ cells per well) in a black, clear bottom 96-well plate coated with 0.1% gelatin (Thermo Fisher Scientific, Waltham, MA, United States) and cultured overnight at 37°C. Compounds were added to the cells ranging from 0 to 1 mM in half-log increments in the presence and absence of 250 μM deferoxamine (DFO), an iron chelator that prevents ferrochelatase from converting protoporphyrin to heme. The addition of DFO allows greater accumulation of the fluorescent intermediate, protoporphyrin, which improves the sensitivity of the assay and helps mimic the effects of the AHPs. Each trial also had three replicates of 0.314 mM allyl isopropyl acetamide (AIA) and 0.314 mM aspirin as the positive and negative controls, respectively. After an 18–24 h incubation, plates were read at an excitation wavelength of 410 nm and an emission wavelength of 625 nm (Figure 1). In a parallel white, solid-bottom 96-well plate, the same conditions were applied to process cytotoxicity via the ATPLite cytotoxicity assay (Figure 2). Background signals were corrected by deducting all fluorescence measurements by the fluorescence produced by DMSO in the absence of DFO. Data and statistical analyses were completed with the software, GraphPad Prism 8.

## Results

In the presence of DFO, the AIA positive control produced fluorescence measurements between 1.6 and 2.1 times greater than the fluorescence measurements from the aspirin negative control in each experiment (Figure 1). The highest readings produced by aspirin, a known non-porphyrogenic drug (NAPOS, 2007), were 0.62 times less than the readings produced by AIA. The highest fluorescence measurements produced by carbamazepine, eslicarbazepine acetate, and phenobarbital were 2.46, 2.08, and 2.35, respectively, times greater than the aspirin control. The fluorescence readings for AIA, aspirin, carbamazepine, and eslicarbazepine acetate begin to decrease at 0.314 and 1 mM likely due to cytotoxicity (Figure 2).

## Discussion

AIA is a known potent porphyrogenic compound that can elicit porphyria even in animals without defects in the heme synthetic pathway, as well as exacerbate the AHPs, so over-production of protoporphyrin to a degree equal to or greater than that produced by 0.314 mM AIA indicates potent porphyrogenicity (Tschudy and Bonkovsky, 1972; Bonkowsky et al., 1973; Bonkovsky et al., 1990; Hahn et al., 1997). Aspirin was selected as the negative control because this drug has been used safely for decades in patients with the AHPs. Thus, protoporphyrin accumulation equal to or below that produced by 0.314 mM aspirin indicates non-porphyrogenicity and suggests drug safety in AHP. Carbamazepine and phenobarbital are already well-known to be porphyrogenic and risky for use in patients with the AHPs (Bonkovsky et al., 1990; Thunell et al., 2007). The results for these two drugs are as expected since they both produced readings greater than the fluorescence reading produced by the AIA control (Figure 1). Eslicarbazepine acetate behaved similarly to carbamazepine in this fluorescence-based screening assay with statistically significant results, which suggests that it too is porphyrogenic.

One recent case report described a patient with focal epilepsy and acute intermittent porphyria (AIP) whose seizures were improved with eslicarbazepine acetate without inducing clinical symptoms of an acute porphyric attack for up to a year. Her urinary PBG levels, however, remained more than 5-fold above the upper limit of the reference range, indicating continuing upregulation of hepatic ALA synthase-1 and risks of further attacks (Herrera-Fortin et al., 2020). It is difficult to make any conclusions without a larger sample size, but our results along with this patient’s elevated urinary PBG levels suggest that eslicarbazepine acetate may be porphyrogenic with potency similar that those of phenobarbital, phenytoin, and carbamazepine, at least in the LMH cell culture system recently described (Ma et al., 2022). Although a causative clinical relationship cannot be established from the *in vitro* findings from this cell culture model to human pathophysiology, the results shown here and previously (Ma et al., 2022) indicate the need for caution and close observation if eslicarbazepine acetate is used in patients with the AHPs. As was done by Dr. Herrera-Fortin and his colleagues, epileptic patients with the AHPs should first be given AEDs that are known to be safer for use in AHPs, such as levetiracetam, topiramate, and benzodiazepines. If these fail to control the seizures, and eslicarbazepine acetate is initiated, patients should be observed closely during the first several months of such therapy, and, if tolerated without exacerbation of the acute porphyria, continue to be regularly monitored for potential porphyric exacerbations (Balwani et al., 2017).

## Data Availability

The original contributions presented in the study are included in the article/supplementary material, further inquiries can be directed to the corresponding author.
